# Development of an Operational Digital Twin of a Locomotive Parking Brake for Fault Diagnosis

**DOI:** 10.1038/s41598-023-45204-1

**Published:** 2023-10-20

**Authors:** Gabriel Davidyan, Jacob Bortman, Ron S. Kenett

**Affiliations:** 1 PHM Laboratory, Department of Mechanical Engineering, Ben-Gurion University of the Negev, P.O. Box 653, 8410501 Beer-Sheva, Israel; 2 KPA Ltd. and the Samuel Neaman Institute for National Policy Research, Technion City, 32000 Haifa, Israel

**Keywords:** Mechanical engineering, Applied mathematics

## Abstract

In recent years, a growing role in digital technologies has been filled by model-based digital twinning. A digital twin produces a one-to-one mapping of a physical structure, operating in the digital domain. Combined with sensor technology and analytics, a digital twin can provide enhanced monitoring, diagnostic, and optimization capabilities. This research harnesses the significant capabilities of digital twining for the unmitigated challenge of fault type classification of a locomotive parking brake. We develop a digital twin of the locomotive parking brake and suggest a method for fault type classification based on the digital twin. The diagnostic ability of the method is demonstrated on a large experimental dataset.

## Introduction

Air brake system is an important component of trains, responsible for speed control in routine operation and emergency braking. In trains, the performance of the air brake system affects the forces in the train, which ultimately affect the driving safety^1,2^. Since building a full-scale test rig is expensive, numerical simulation techniques are often used to study air brake systems. Empirical models are among the most commonly used approaches, which include lookup table models and fitting formula models^3^. These models are suitable for practical applications that do not require detailed knowledge of the air brake system, such as signaling design and braking distance calculation. They are based on measured data and provide equivalent constant values for the braking forces^4–9^. However, to improve the system and predict its characteristics, dynamic models based on physical principles are required. These models, which are widely used in practice, can simulate the air brake system by considering the energy exchange of the system and the air leakage of the pipe^10–14^. Despite their usefulness, current dynamics models have not produced robust fault type classification algorithms because there are significant differences between simulated and actual operating conditions^15^.

Digital twin (DT) refers to a virtual representation of physical systems that has various applications, such as performance optimization^16,17^. DTs are developed through mathematical models and simulations and are continuously updated with real-time data from sensors of the physical systems^18,19^. DTs are increasingly being used across different industries, including manufacturing, transportation, energy, and healthcare^20,21^. A key advantage of DTs is their ability to make accurate and reliable predictions about a system's behavior, enabling better utilization of the physical system^22,23^. For instance, a DT of a factory could be used to predict the output of each production line under different operating conditions and identify possible inefficiencies^24^. In a related study^25^, a DT was created by virtually modeling brake pads in real time, which provided information about their wear and tear, improving vehicle safety and operational efficiency. Similarly^26^, addressed the development of a digital twin for the automotive braking system, using physics-based modeling techniques to predict brake pad wear under various conditions. In this approach, different modeling paradigms, from 0-D to 3-D, were merged into an integrated system model, further demonstrating the versatility and robustness of digital twins in modern engineering applications.

However, implementing DTs can be difficult, as it is challenging to guarantee that the DT accurately represents the system^27^. One possible solution is to use machine-learning algorithms to enhance the accuracy of mathematical models using sensor data^28^. These algorithms can address the differences between simulation and reality^29,30^ and, hence, provide DT improvements.

In this study, a model-based DT approach for classifying various faults that may occur in a locomotive parking brake is proposed and validated by experiments. The DT is based on a physical model of the parking brake and is optimized using a machine learning approach. The DT of the locomotive parking brake is continuously updated to generate possible fault conditions that approximate those of the actual system. The study has three major contributions:

1. Development of a DT of a locomotive parking brake.
2. Development of a robust diagnosis of various faults in a locomotive parking brake by estimating the internal latent physical variables within a DT and training of a learning model on the residuals signals and model based estimated internal variables.
3. Show how deep physical understanding and model-based DT can improve the diagnostic capabilities of complex systems, especially in scenarios where machine learning algorithms alone may not be sufficient.

The study is divided into six sections. Section “Theoretical background” provides a theoretical background and introduces the locomotive parking brake and the physical model DT, Section “Experimental validation of the proposed parking brake model” validates the physical model DT using experimental data, Section “The proposed algorithm” presents the new algorithm based on DT, and Section “Fault diagnosis” demonstrates the algorithm using experimental data. The study is summarized in Section “Summary and conclusions”.

## Theoretical background

### Parking brake working principle

**Figure 1:**
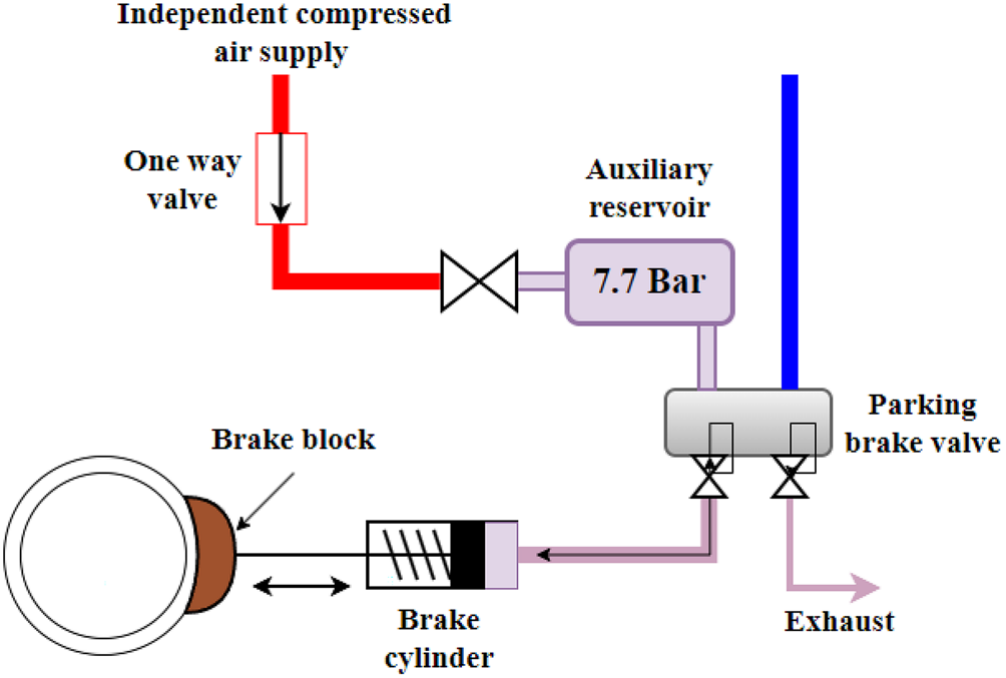
Schematic diagram of the parking brake system.

The JT42 locomotive is equipped with a spring-loaded brake. Four of the eight brake blocks are equipped with load springs, as shown schematically in Fig. 1. These four brake blocks contain in the rear part of their housing the following main components: a piston, a load spring, and a manual release device. The cylinder part equipped with the load spring has an independent compressed air connection; the piston chamber is hermetically isolated from the service brake chamber and is additionally separated by an atmospheric sluice. In the released state, the loading spring cylinder is filled with compressed air, and the piston and connecting rod are retracted against the resistance of two springs until no force is applied against the piston base of the brake shoe (in the front part of the cylinder). In this position, the springs of the parking brake are loaded and energy is stored in them. In the applied (brake operative) state, the piston of the spring storage cylinder chamber is empty of compressed air. The force of the springs press on the piston of the service brake cylinder via the connecting rod, which presses the brake pads onto the wheel.

To apply or release the parking brakes, an electrical switch is located in each driving cab. A relay controls the one-way solenoid valve and allows compressed air from the main reservoir line to flow into the spring chamber through a pressure-reducing valve set at 7.7 bar and through an orifice in the parking brake valve. This compresses the loading spring in the spring chamber and the parking brakes remain in the non-operated mode. A healthy parking brake should be completely released after 25 s. By switching off the solenoid valve, the compressed air is released from the spring chamber so that the brake shoes are driven against the wheel by the force of the spring. For more detailed information on the braking system, see^31,32^.

### Parking brake model

Physical systems must first be represented in terms of a mathematical equation for each machine before any particular analysis can be performed for those particular systems. Mathematical modeling is required before any control over the systems can be explored. In general, the modeling of each system can be created either from physical derivatives or experimental data measured on the real system. This paper presents nonlinear mathematical modeling of a JT42 locomotive parking brake based on readily accessible physical derivatives. The mass flow rate, pressure dynamics, and equation of motion were derived from previous research^30^.

The compressible mass flow rate, $\dot{m}$ through a valve orifice, can be described as^1^;

1 $$\dot{m}\left( {P_{u} ,P_{d} } \right) = \left\{ \begin{gathered} P_{u} C_{f} C_{1} \frac{1}{\sqrt T }, \frac{{P_{d} }}{{P_{u} }} < P_{cr} \hfill \\ P_{u} C_{f} C_{2} \frac{1}{\sqrt T }, \frac{{P_{d} }}{{P_{u} }} > P_{cr} \hfill \\ \end{gathered} \right.$$

where ${P}_{u},{P}_{d}$ are the upstream and downstream pressure respectively, ${C}_{f}$ is the non-dimensional discharge, *T* temperature and *k* is the specific heat ratio, and $\dot{m}\left({P}_{u},{P}_{d}\right)$ is the mass flow rate.

The constants ${C}_{1}$ and ${C}_{2}$ are given by;

2 $${C}_{1}=\sqrt{\frac{k}{R}{\left(\frac{2}{k+1}\right)}^{\frac{k+1}{k-1}}}$$

3 $${C}_{2}=\sqrt{\frac{k}{R}\left(\frac{2}{k+1}\right)}$$

where $R$ is the gas constant, ${P}_{cr}$ is a critical pressure related to the ratio between downstream pressure ${P}_{d}$ and upstream pressure ${P}_{u}$. The value of ${P}_{cr}$ can be expressed as;

4 $${P}_{cr}={\left(\frac{2}{k+1}\right)}^{\frac{k}{k-1}}$$

The upstream and downstream pressures of the cylinder are different, depending on whether the cylinder chamber is charging or discharging, according to the following functions:

5 $${\dot{m}}_{in}=\dot{m}\left({P}_{s},{P}_{chamber}\right)$$

6 $${\dot{m}}_{out}=\dot{m}\left({P}_{chamber},{P}_{a}\right)$$

where ${\dot{m}}_{in}$, ${\dot{m}}_{out}$ are the mass flow rate into and out of cylinder chamber A respectively, ${P}_{chamber}$ is the pressure inside the cylinder chamber, ${P}_{s}$ is the supply pressure and ${P}_{a}$ is the ambient pressure.

Equation (5) represents the charging process, in which the pressure in the reservoir is considered to be upstream and the pressure in the cylinder chamber is downstream. For the discharging process, represented by Eq. (6), the pressure in the chamber is the upstream pressure and the ambient pressure is the downstream pressure. The flow condition can be classified as either choked flow or under-choked flow, depending on the downstream pressure ${P}_{d}$ and the upstream pressure ${P}_{u}$ of the orifice. The flow condition is considered to be choked flow when $\frac{{P}_{d}}{{P}_{u}}$ is less than the critical pressure ratio ${P}_{cr}$. Assuming that the gas is perfect and the process is considered adiabatic, the rate of pressure change in a pneumatic chamber can be expressed as follows;

7 $$\frac{\partial }{\partial t}P\left(t\right)=\frac{RTk}{V(t)}\left(\frac{\partial }{\partial t}{m}_{in}\left(t\right)-\frac{\partial }{\partial t}{m}_{out}\left(t\right)\right)-\frac{kP\left(t\right)}{V(t)}\frac{\partial }{\partial t}V\left(t\right)$$

where $P\left(t\right)$ is the pressure inside the chamber, $\frac{\partial }{\partial t}P\left(t\right)$ rate of change in pressure inside chamber, $\frac{\partial }{\partial t}{m}_{in}\left(t\right)$ inlet mass flow rate to chamber, $\frac{\partial }{\partial t}{m}_{out}\left(t\right)$ outlet mass flow rate from the chamber,$V\left(t\right)$ is the volume of the chamber, and $\frac{\partial }{\partial t}V\left(t\right)$ rate of change in volume of the chamber.

The origin of the piston placement can be chosen either from one side at the end of the stroke or in the middle of the stroke. By choosing the origin on one side at the end of the stroke, the volume of the chamber can be expressed as follows:

8 $$V\left(t\right)={A}_{p}x(t)$$

where $x(t)$ is the piston displacement and ${A}_{p}$ is the area of the piston.

By differentiating Eq. (8), the rate of change of the volume of the chamber is obtained as follows:

9 $$\dot{V}\left(t\right)={A}_{p}\frac{\partial }{\partial t}x(t)$$

Substituting Eqs. (8), (9) into Eq. (7) gives the rate of pressure change in the chamber of the pneumatic cylinder:

10 $$\frac{\partial }{\partial t}P\left(t\right)=\frac{RTk}{{V}_{0}+{A}_{p}x(t)}\left(\frac{\partial }{\partial t}{m}_{in}\left(t\right)-\frac{\partial }{\partial t}{m}_{out}\left(t\right)\right)-\frac{kP\left(t\right)}{{V}_{0}+{A}_{p}x(t)}{A}_{p}\frac{\partial }{\partial t}x(t)$$

where $x(t)$ is the piston position,$\frac{\partial }{\partial t}x(t)$ is the rate of change of piston position, and ${V}_{0}$ is the inactive volume at the end of stroke and admission port.

The equation of motion for the piston rod, including the mass and friction effects of the pneumatic cylinder, can be expressed by Newton's second law as follows:

11 $${M}_{p}\frac{{\partial }^{2}}{{\partial }^{2}t}x\left(t\right)={P}_{A}{A}_{p}-{F}_{f}-{P}_{a}{A}_{r}-Kx\left(t\right)$$

where ${M}_{p}$ is the mass of the piston rod, ${F}_{f}$ is the friction force, ${P}_{a}$ is the atmospheric pressure, ${A}_{r}$ is the cross-sectional area of the piston rod and $K$ is a constant factor characteristic of the spring.

The LuGre friction model proposed by Liu et al.^33^ is considered in this study. The frictional force, $F_{f}$, the dynamics of the internal state ($z$), and the Stribeck effect function $g(v)$ are given in Eqs. (12), (13), and (14), respectively. From these equations, the relationship between velocity and friction force for the steady-state of motion can be derived as Eq. (15):

12 $${F}_{f}={\sigma }_{0}z+{\sigma }_{1}\frac{\partial }{\partial t}z+Bv$$

13 $$\frac{\partial }{\partial t}z=v-{\sigma }_{0}\frac{|v|}{g(v)}z$$

14 $$g\left(v\right)=\frac{{F}_{c}+({F}_{s}-{F}_{c}){e}^{-{\left(\frac{v}{{v}_{s}}\right)}^{2}}}{{\sigma }_{0}}$$

15 $${F}_{f}={\sigma }_{0}g\left(v\right)sgn\left(v\right)+Bv={F}_{c}sgn\left(v\right)+\left({F}_{s}-{F}_{c}\right){e}^{-{\left(\frac{v}{{v}_{s}}\right)}^{2}}sgn\left(v\right)+Bv$$

where $\sigma_{0}$ is a stiffness coefficient, $\sigma_{1}$ is a damping coefficient, B is the viscous friction, $F_{c}$ is the Coulomb friction, and $F_{s}$ is the static friction.

## Experimental validation of the proposed parking brake model

The parking brake DT is configured to simulate three typical fault types, including cylinder leakage, blocked upstream pressure, and low upstream pressure. A total of five working states of the parking brake system are simulated using a DT, including one health state, two types of single fault states, and two types of composite fault states as shown in Table1. The model parameters were calculated assuming the following; the total heat energy of the gas does not change during compression. The specific heat coefficient for air is $k=1.4$. The value of the gas constant $R$, temperature $T$ and ambient pressure Po at a relative humidity of 65% are $288\;{\text{J}}/{\text{kgK}},$ $293.15\;{\text{K}}$ and $100\;{\text{kPa}}$, respectively (Standard ISO 6358). This gives constants *C*_1_ and *C*_2_ of $0.040418$ and $0.156174$, respectively, which were calculated using Eq. (3). Equation (4) is used to set the critical pressure ratio *p*_cr_ to $0.528$. The pressure after the pressure-reducing valve was set at $7.7\;{\text{bar}}$. The cylinder bore diameter is $0.1\;{\text{m}}$.

**Table 1:** Working conditions of parking brake.

Working states of the parking brake	Total number of samples
Normal state	500,000
Air leakage	500,000
Blocking due to an incorrectly set orifice	500,000
Cylinder leakage & blocking	500,000
Low upstream pressure	500,000

**Figure 2:**
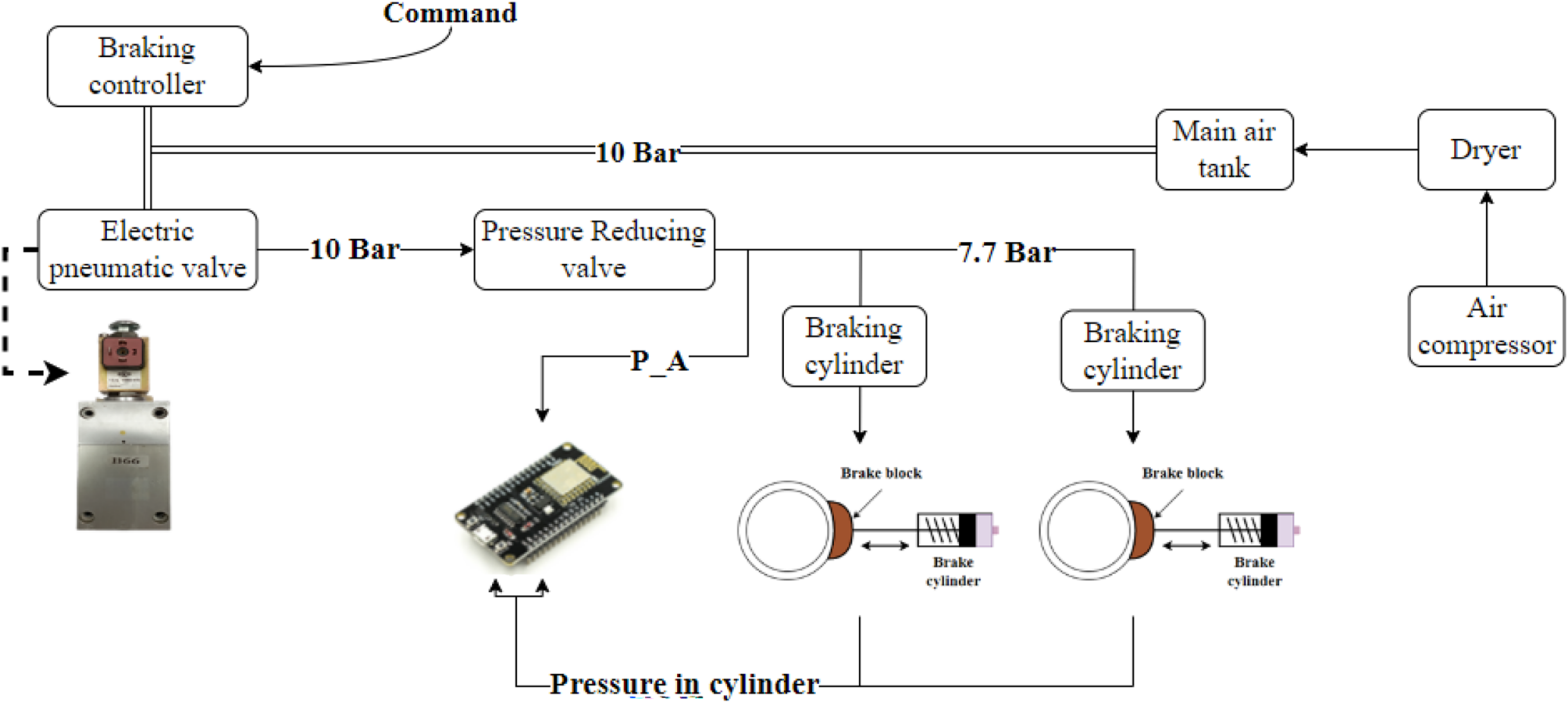
Schematic diagram of the parking brake system and experimental setup.

To verify the proposed mathematical model, a series of experiments was conducted in which the pressure in the cylinder chamber and after the pressure-reducing valve was were measured using piezoelectric absolute pressure transducers, as can be seen in Fig. 2.

**Figure 3:**
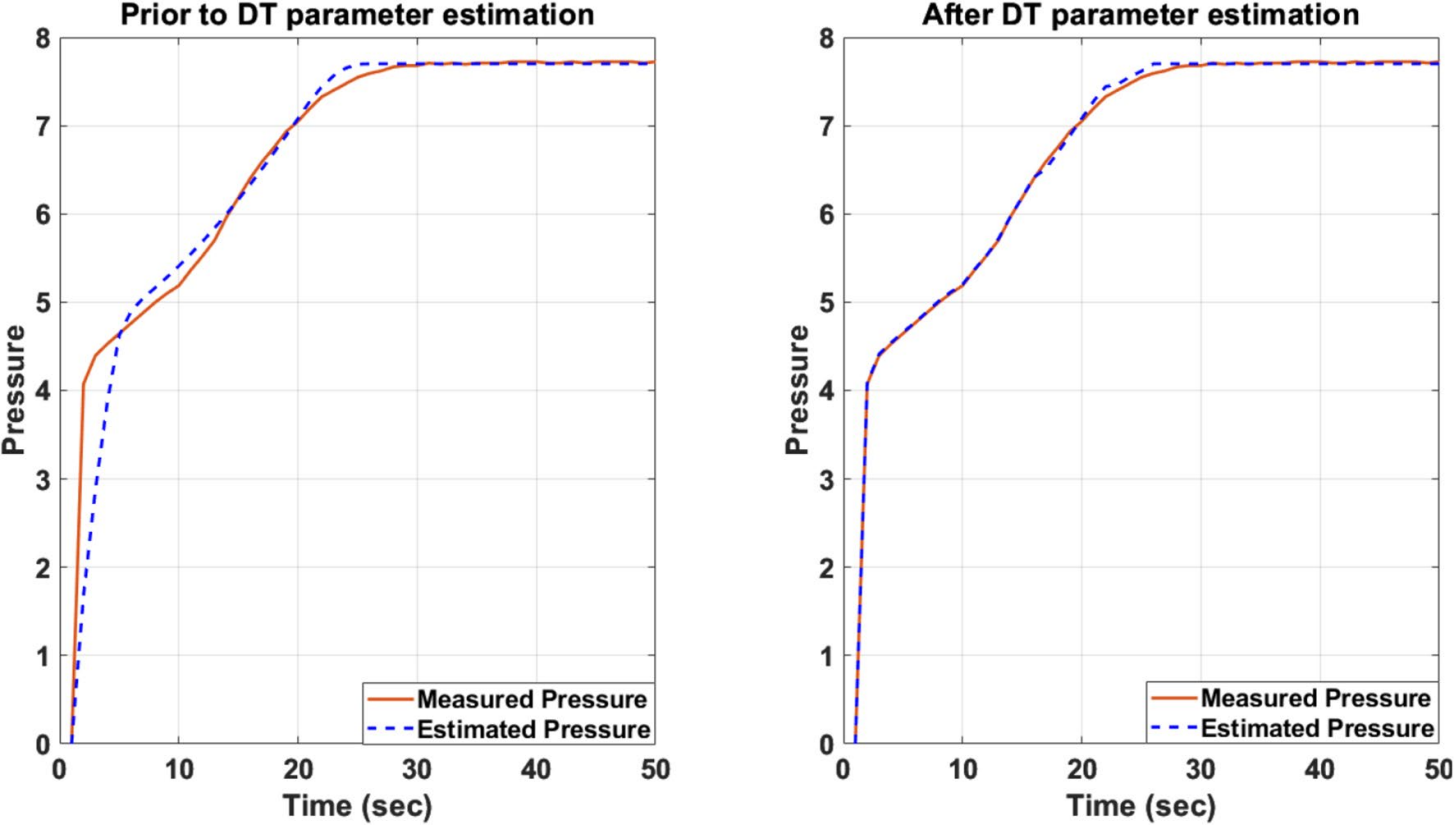
Model (blue curve) and experimental (red curve) results for the pressure in the cylinder chamber of healthy parking brake.

The experiments were conducted as follows: First, the parking brake was applied (i.e. the pressure in the cylinder chamber is 0 bar), and then the parking brake was released using the electric switch in the driver's cab. Figure 3 shows both the model and experimental results for the pressure in the cylinder chamber. There is close agreement between the theoretical and experimental curves, with very good amplitude agreement. Figure 3 also shows a time delay between the valve command (0 s) and the corresponding pressure output. This phenomenon could be due to the connecting pipes and the dynamics of the actuator valve. In the first stage the pressure in the cylinder chamber rises rapidly to 4.07 bar. Then, as the piston begins to move, the rate of change of pressure decreases as the volume of the cylinder chamber increases. Finally, when the piston reaches its end position (25 s), the pressure rate of change is zero and the pressure in the cylinder chamber is equal to the pressure of the pressure-reducing valve, i.e. 7.7 bar. The results show that the DT simulates the parking brake with great accuracy, which reflects the quality of the mathematical procedures used.

**Figure 4:**
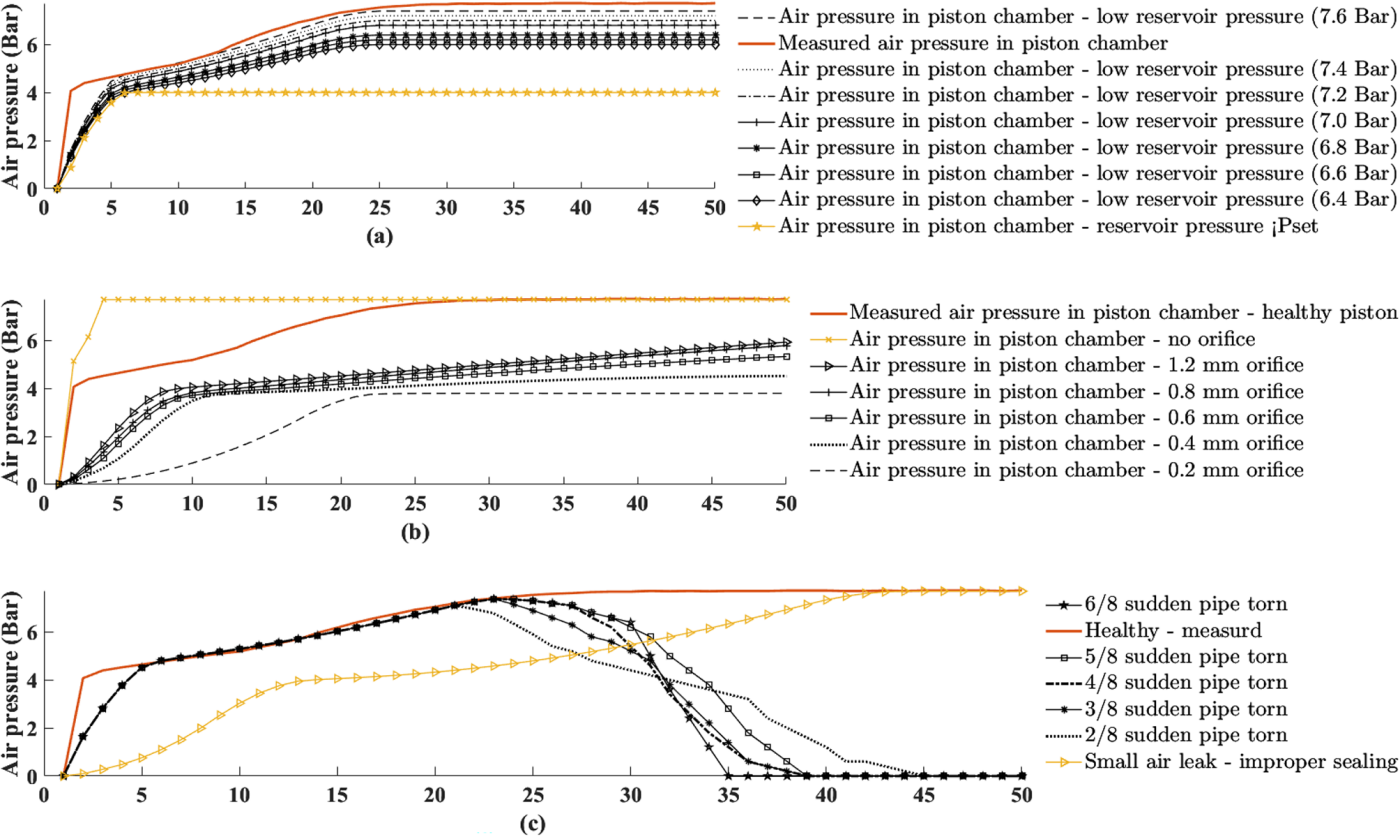
Model results show the response of the pressure in the cylinder chamber for different faults compared with healthy experimental results (red curve). (**a**) low up-stream pressures, (**b**) different sizes of the orifice, (**c**) sudden air leak in up-stream pressure.

Based on the verified DT model, different failure states of the parking brake system can be simulated by adjusting some parameters of the model. Figure 4a shows the results obtained when an experiment was performed with an upstream pressure of 7.7 bar illustrates a healthy parking brake system (red curve). The figure also shows the simulated curves of the pressure in the cylinder chamber obtained using the DT for a scenario where the outlet (upstream) pressure of the pressure reducer is lower than 7.7 bar. In all scenarios excluding the case where the upstream pressure is lower than the set pressure (i.e. 4.027 bar, yellow), there is close agreement between shape of the curves. The results in Fig. 4a shows that in the case where the upstream pressure is lower than the set cylinder pressure, there is a rapid increase in the pressure inside the cylinder up to the set pressure. From this point it remains constant (4.027 bar). This means that the chamber volume does not change because the force acting on the piston is too small (the piston remains in place, i.e. the parking brakes do not release). Figure 4b shows the results of an experiment in which the upstream pressure for both DT and the real parking brake system (red curve, illustrates a healthy parking brake system) was set to 7.7 bar. In this scenario, blocking of the upstream pressure due to an incorrectly set orifice was simulated using the DT. Figure 4b shows that the pressure in the cylinder chamber quickly rises (3 s) to the upstream value (7.7 bar) when no orifice was inserted into the parking brake valve outlet (yellow). In this case, the piston moves violently towards its end position. This situation can cause severe damage to various parts of the parking brake or even to the entire mechanism. In contrast, too small and orifice can cause a significant delay in releasing the parking brake. The results in Fig. 4b show that the rate of change of pressure in the cylinder chamber using an orifice of 0.4 mm (dotted curve), for example, is small compared to the healthy condition (red curve, illustrates a healthy parking brake system). In this case, the pressure in the cylinder chamber reached the set pressure value (4.027 bar) only after 50 s, causing a significant delay in the parking brake release application. Figure 4c shows the results of an experiment in which the upstream pressure drops due to leakage in the cylinder or in the connecting pipes. The figure shows the different rates of pressure drop in the cylinder chamber found experimentally and simulated using DT compare to a healthy parking brake system (red curve). Some curves represent a rapid pressure drop, simulating a severe leak typical of a torn pipe, while other curves (yellow) represent a pressure drop reminiscent of a small leak due to improper sealing. Figure 4c shows that a severe leak due to a torn pipe causes the air pressure in the cylinder chamber to drop rapidly, causing the piston to move uncontrollably toward its initial position. Such a movement can severely damage the brake pad. On the other hand, a leak resulting from improper sealing can be overcome by the supplying pressure to the cylinder. However, this results in a considerable delay in releasing the parking brake.

To evaluate the capacities of the DT, the relative Normalized Mean Squared Error (NMSE) between the measured and simulated pressures in the cylinder chamber at each time point was calculated as follows:

16 $${\text{NM}}S{\text{E }} = 100\% \times \sqrt {\frac{1}{N}\mathop \sum \limits_{i = 1}^{N} \frac{{\left( {{\text{P}}_{Ai} - \widehat{{P_{Ai} }}} \right)^{2} }}{{{\text{P}}_{{{\text{A}}i}}^{2} }}}$$

where *N* is the total number of time points, ${\mathrm{P}}_{A}$ is the measured signal, $\widehat{{P}_{A}}$ is the DT simulated signal and $i$ the index represents the time points.

**Figure 5:**
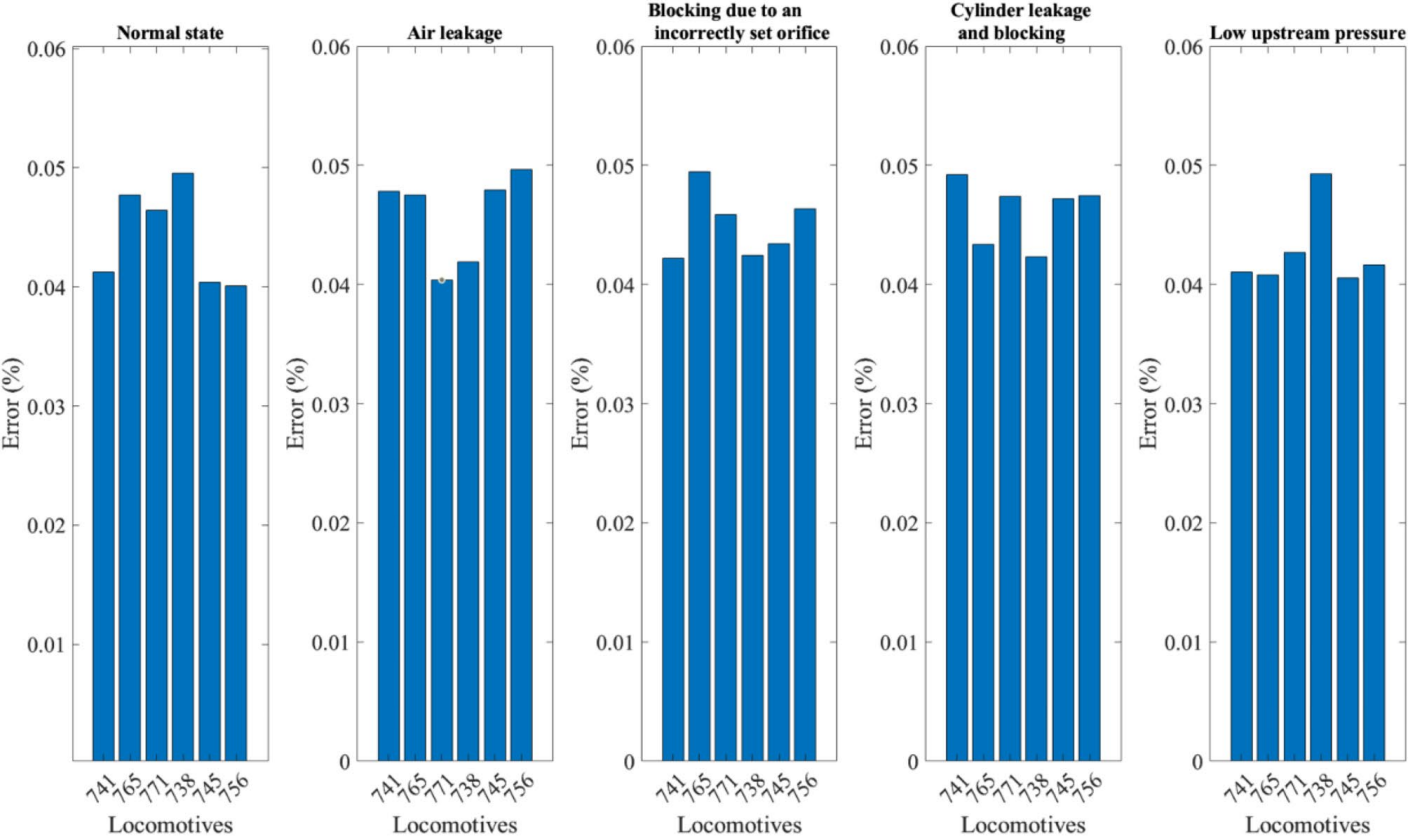
NMSE index showing the mean square error between the experimental and simulated pressures in the cylinder chamber for different scenarios for six locomotives.

To calculate the NMSE, the same experiments simulating the different scenarios (see Table 1) were performed with six locomotives. Figure 5 shows the NMSE comparing each DT simulated scenario and the corresponding measured scenario. For example, the leftmost column shows the NMSE index between the measured and simulated air pressure in the cylinder chamber at normal state. The highest error was calculated for the experiment simulating an air leak in the cylinder together with an air blockage. The relatively high error can be explained by the relative complexity of the experiment and the sensitivity of the different parking brake valves. However, Fig. 5 shows that the mean square error is relatively small (highest value, approx. 5%) for all simulated scenarios, reflecting the quality of the DT.

## The proposed algorithm

**Figure 6:**
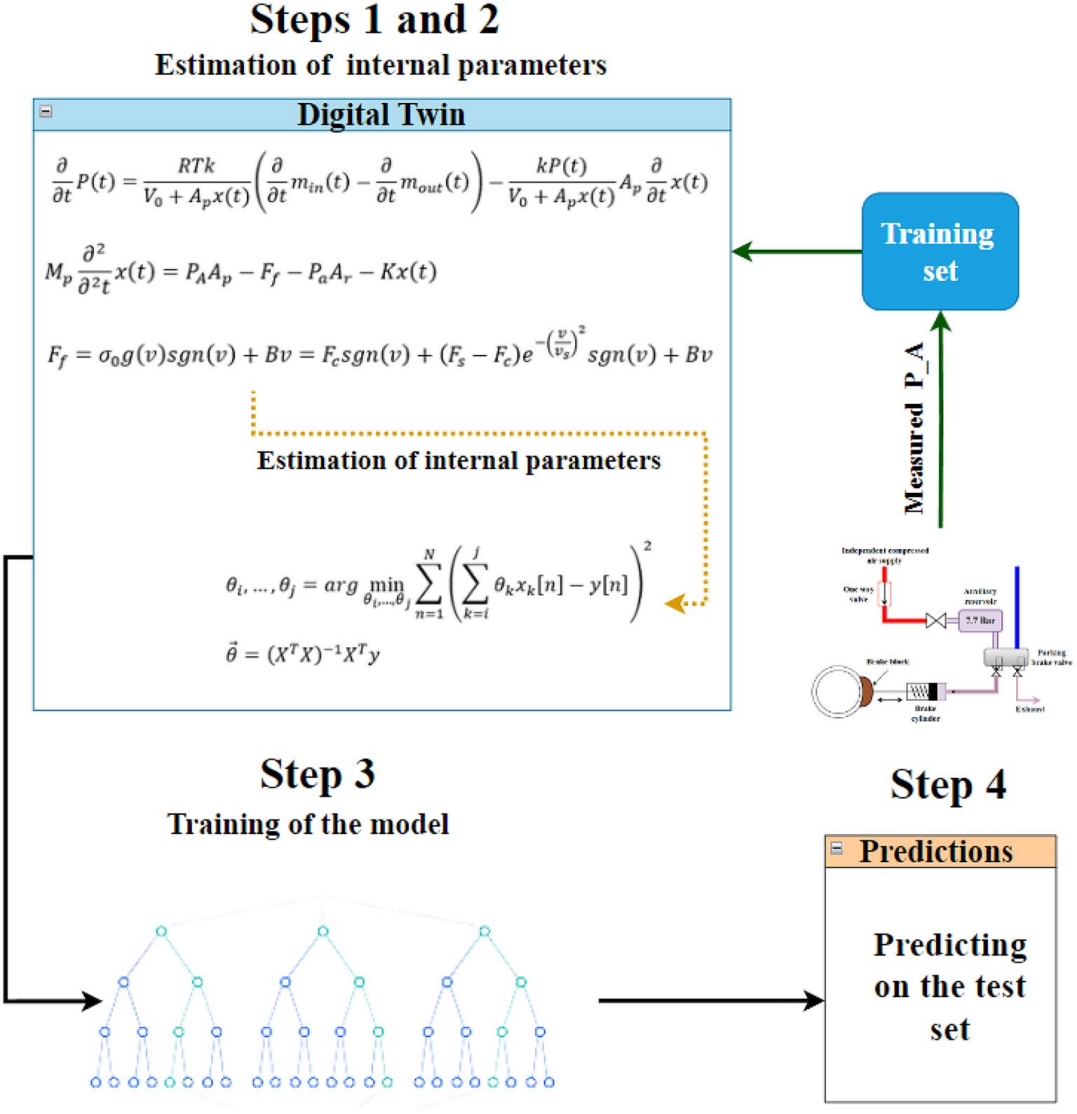
The new suggested algorithm for machine fault diagnosis using a DT described in Section “The proposed algorithm”.

The proposed algorithm is integrated in the locomotive DT. It consists of four steps, illustrated in Fig. 6.

The pressure in the parking brake cylinder (marked by ${P}_{A}$) was routinely measured on the tested locomotives, as can be seen in Fig. 6. In the current study, the internal latent physical variables ($K, B, {\sigma }_{0}$ and ${\sigma }_{1}$) are estimated by solving a least squares problem, where the variables minimize the constraints presented in Eq. (17). This is achieved in Eq. (18), also known as a least squares estimation:

17 $${\theta }_{i},\dots ,{\theta }_{j}=arg\underset{{\theta }_{i},\dots ,{\theta }_{j}}{\mathrm{min}}\sum_{n=1}^{N}{\left(\sum_{k=i}^{j}{\theta }_{k}{x}_{k}\left[n\right]-y\left[n\right]\right)}^{2}$$

18 $$\vec{\theta }={\left({X}^{T}X\right)}^{-1}{X}^{T}y$$

where ${x}_{k}\left[n\right]$ represents the corresponding values of the internal parameters ${\theta }_{k}$ in the coordinate $n$ $\left[ {{\text{e}}.{\text{g}}.,\;{\text{for}}\;\theta_{k} \;{\text{in}}\;{\text{time}}\;t\;{\text{the}}\;{\text{corresponding}}\;{\text{value}}\;{\text{is}}\;P_{A} \left( {\frac{t}{\Delta t}} \right)} \right]$, $y\left[n\right]$ represents the measured parameters in the coordinate $n$ and $X$ is the matrix of ${x}_{k}\left[n\right]$ with $N$ rows and $j-i+1$ columns.

The internal variables (${\theta }_{1}, {\theta }_{2}, {\theta }_{3}$ and ${\theta }_{4}$) can be used to calculate the process coefficients presented in Eq. (19):

19 $$K={\theta }_{1}, B={\theta }_{2}, {\sigma }_{0}={\theta }_{3}, {\sigma }_{1}={\theta }_{4}$$

For each Real Twin (RT) (i.e., physical measurement ${P}_{A}$) in the training set, an individualized DT is generated by estimating the internal variables (${\theta }_{1}-{\theta }_{4})$. These variables are estimated by the least squares method presented in Eq. (17).

Based on the internal estimated parameters, a model of Deep Neural Network (DNN) is trained on the estimated parameters where, at first, the training set is divided into 80% training and 20% validation. The DNN model uses the sigmoid activation function in the input and hidden layers, while the Softmax function is used in the output layer. The selected optimization algorithm was Adam with an initial learning rate of 0.001 and the loss was categorical cross entropy. The DNN model included an input layer, eight hidden layers with eight neurons each, and one output layer. The number of epochs was regulated using early stopping based on three followed unimproved loss on the validation set.

For each Real Twin (RT) (i.e., physical measurement ${P}_{A}$) in the test set, an individualized DT is generated by estimating the internal variables (${\theta }_{1}-{\theta }_{4})$. These variables are estimated by the least squares method presented in Eq. (17). The trained model is used to predict the classes of the test set.

## Fault diagnosis

**Figure 7:**
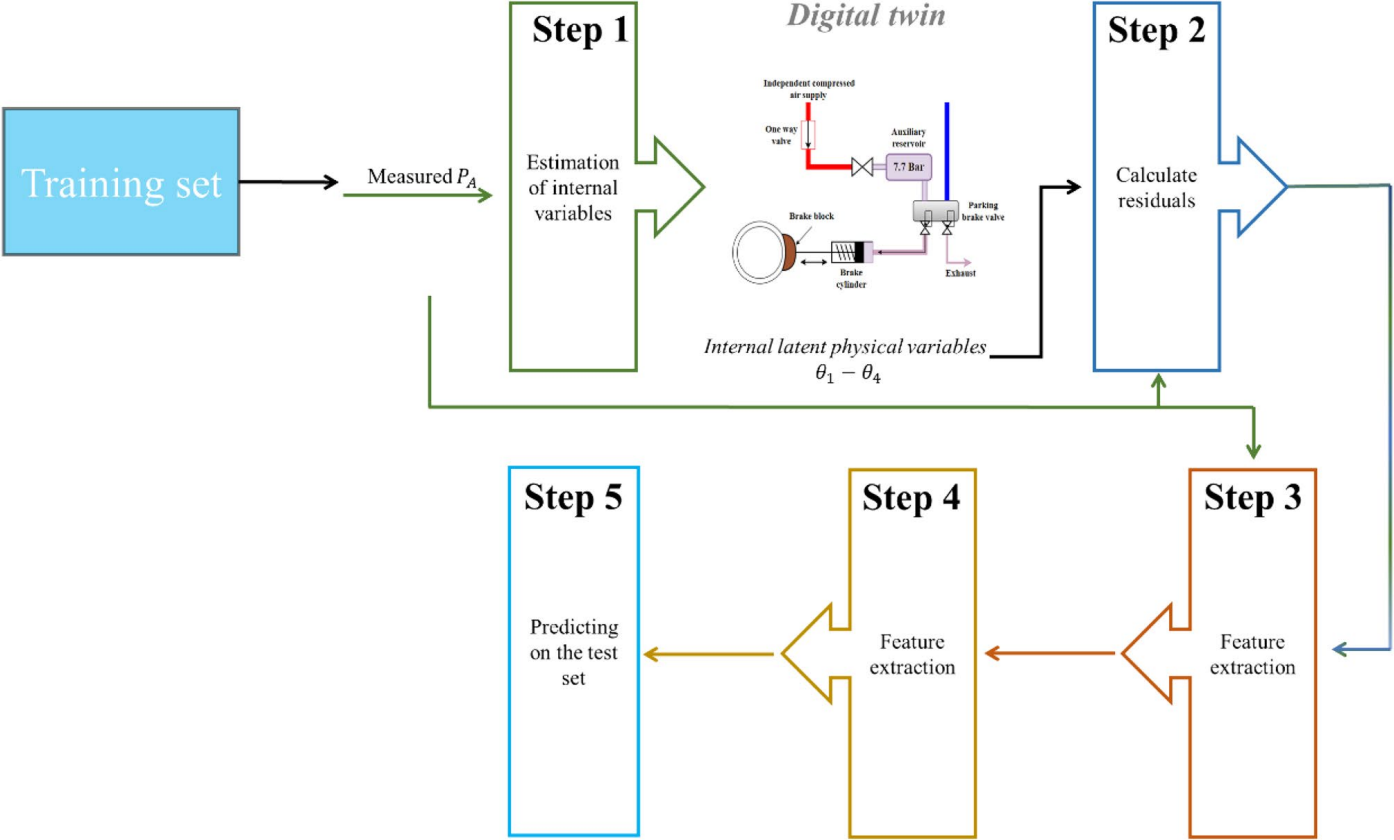
The two other algorithms use as comparison to the new suggested algorithm described in Section “Experimental validation of the proposed parking brake model”.

The new algorithm described in Section “Experimental validation of the proposed parking brake model” is tested and compared with two other algorithms:

1. A regular machine-learning algorithm using a DNN model consists of Steps 3, 4, and 5 described in Fig. 7. The regular machine-learning algorithm was trained with 21 different possible features: Mean, Variance, Maximum, Kurtosis, standard deviation, skewness, Absolute Sum etc., of each RT’s features extracted directly from the measured signals, i.e., ${P}_{A}$. The DNN model is trained on these extracted features, as described in Fig. 7. This algorithm does not use the DT.
2. A simple residual algorithm which uses Steps 1, 2, 3, 4 and 5 (including the DT) described in Fig. 7. For each RT in the training set, a DT is generated by estimating the internal variables, as explained in Fig. 7. Based on the internal estimated parameters, the DT calculates the residuals. From each residual, 21 features are extracted: mean, variance, maximal value, kurtosis, absolute sum etc. A model of deep neural network is trained on these extracted features, as described in Fig. 7.

The comparison process between the new algorithm and the regular machine-learning algorithm demonstrates the contribution of the DT concept, while the comparison to the simple residual algorithm demonstrates the contribution of the internal variables estimation. These three algorithms, i.e., the new algorithm described in Section “Experimental validation of the proposed parking brake model”, the regular machine-learning algorithm, and the simple algorithm, were tested on an experimental dataset containing a 5500 RT training set and a 250 RT test set. An example of the measurements of an RT in the training set is depicted in Fig. 3.

**Figure 8:**
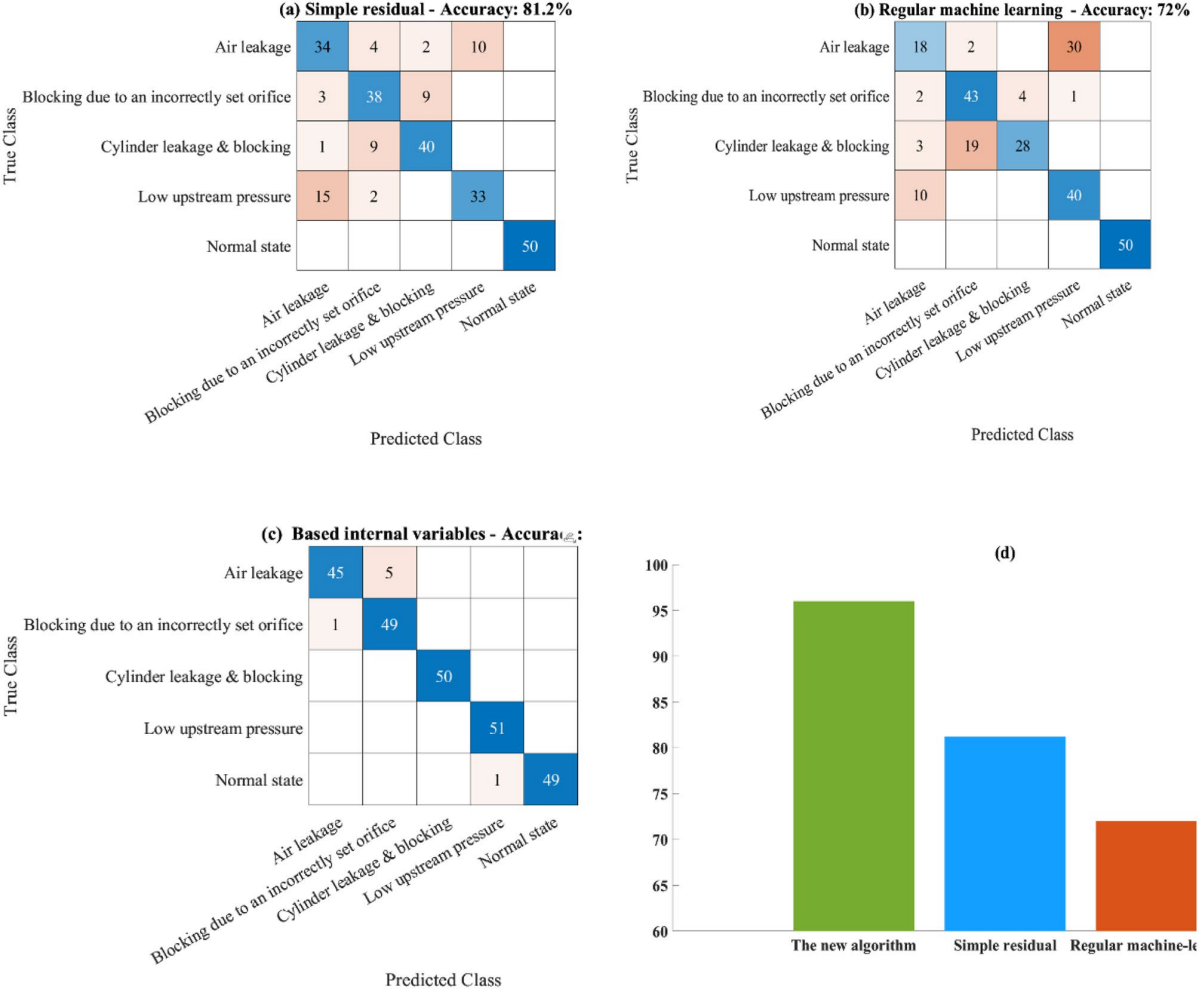
Results of the new suggested algorithm. (**a**) simple residual (the new algorithm with the DT), (**b**) regular machine-learning algorithm without the DT and (**c**) the new suggested algorithm. (**d**) The summarized accuracies of (**a**–**c**).

The results of the three tested algorithms are presented in Fig. 8 on 250 test examples, 50 from each condition. Each table in Fig. 8a–c presents the confusion matrix after applying the tested algorithm on the test set, which contains 50 examples of each category (overall, 250 examples). As can be seen in Fig. 8d, the new algorithm achieved a significant improvement from an accuracy of 72–96. Furthermore, the use of the residuals helps to improve the accuracy from 72 to 81.2, as can be seen in Fig. 8d. This result demonstrates the ability of the DT to improve diagnosis by incorporating physical knowledge of the system.

## Summary and conclusions

In this study, a DT algorithm for diagnosing faults in a locomotive parking brakes is presented. The algorithm comprises four steps, including estimation of the internal DT variables, training of a deep neural network, and prediction. The algorithm was tested with a dataset of 5500 training RTs and 250 test RTs. The results show a significant improvement in accuracy from 72 to 96 compared to a traditional machine-learning algorithm. It is shown that the DT improves diagnosis by incorporating physical knowledge about the system. The described method for model-based fault detection and diagnosis for locomotive parking brakes is based on standard measurement of the pressure in the parking brake cylinder. A key advantage of the described DT based fault detection method is that it uses a physically-derived parking brake model. Therefore, it is applicable to a wide range of operating points and directly transferable to other parking brakes. In addition, its symptoms are usually easy to interpret and understand. The model-based DT approach presented here provides an application of engineering performance that goes beyond traditional engineering design. In addition to impacting train operation, it can also play a role in improving maintenance activities in which maintenance failures are effectively diagnosed and corrected. Ultimately, the most important benefit of the DT is its impact on customer experience and operating costs. This paper demonstrates the value of performance-focused engineering where real-time performance provides inputs to an adaptable system packaged in a digital layer—the DT.

## Data Availability

The datasets generated and/or analysed during the current study are not publicly available hence all data and codes used in this, subject to approval by Israel Railways, but are available from the corresponding author on reasonable request.

## Abbreviations

DT: Digital twin, a digital representation of the physical parking brake
RT: Real twin, physical measurement from the parking brake
DNN: Deep neural network
NMSE: Normalized mean squared error
